# Study on deterioration mechanism of soil in Zhouqiao site under salinization

**DOI:** 10.1038/s41598-022-15802-6

**Published:** 2022-07-07

**Authors:** Jianwei Yue, Huijie Gao, Limin Zhao, Qingmei Kong, Xiangchun Xu, Zifa Wang, Ying Chen

**Affiliations:** 1 School of Civil Engineering and Architecture, Henan University, Kaifeng, 475004 China; 2 Key Laboratory for Restoration and Safety Evaluation of Immovable Cultural Relics in Kaifeng City, Kaifeng, 475004 China; 3 Yellow River Civilization and Sustainable Development Research Center, Henan University, Kaifeng, 475004 China

**Keywords:** Engineering, Materials science

## Abstract

Alkalinity production is one of the most typical and widespread salinization hazards on the Loess Plateau. Based on the characterization of typical flooding sites and the results of salt monitoring, this study investigates the deterioration mechanism of salinization on Zhouqiao site. The orthogonal test was used to simulate the effects of different concentrations of MgSO_4_, NaCl and CaCl_2_ under natural conditions on the quality change, salt analysis out location, surface phenomenon, strength and electrical conductivity of the soil at the Zhouqiao site, and to make a preliminary analysis on the mechanism of saline deterioration of the site soil. The results show that the soil column mass increased significantly under the action of salt, and the rate of salt absorption in the soil column decreased when the critical value was reached, and the critical values were different under the action of different kinds of salts. The rate of salt analysis is also influenced by the salt concentration and the number of cycles, which gradually increases with the increase of salt concentration and the number of cycles. The nominal strength of the soil column with the number of cycles, but occasionally increases. The conductivity increases with the number of cycles, and the magnitude distribution of the conductivity of the soil column under the action of different salts is not exactly the same.

## Introduction

**Figure 1:**
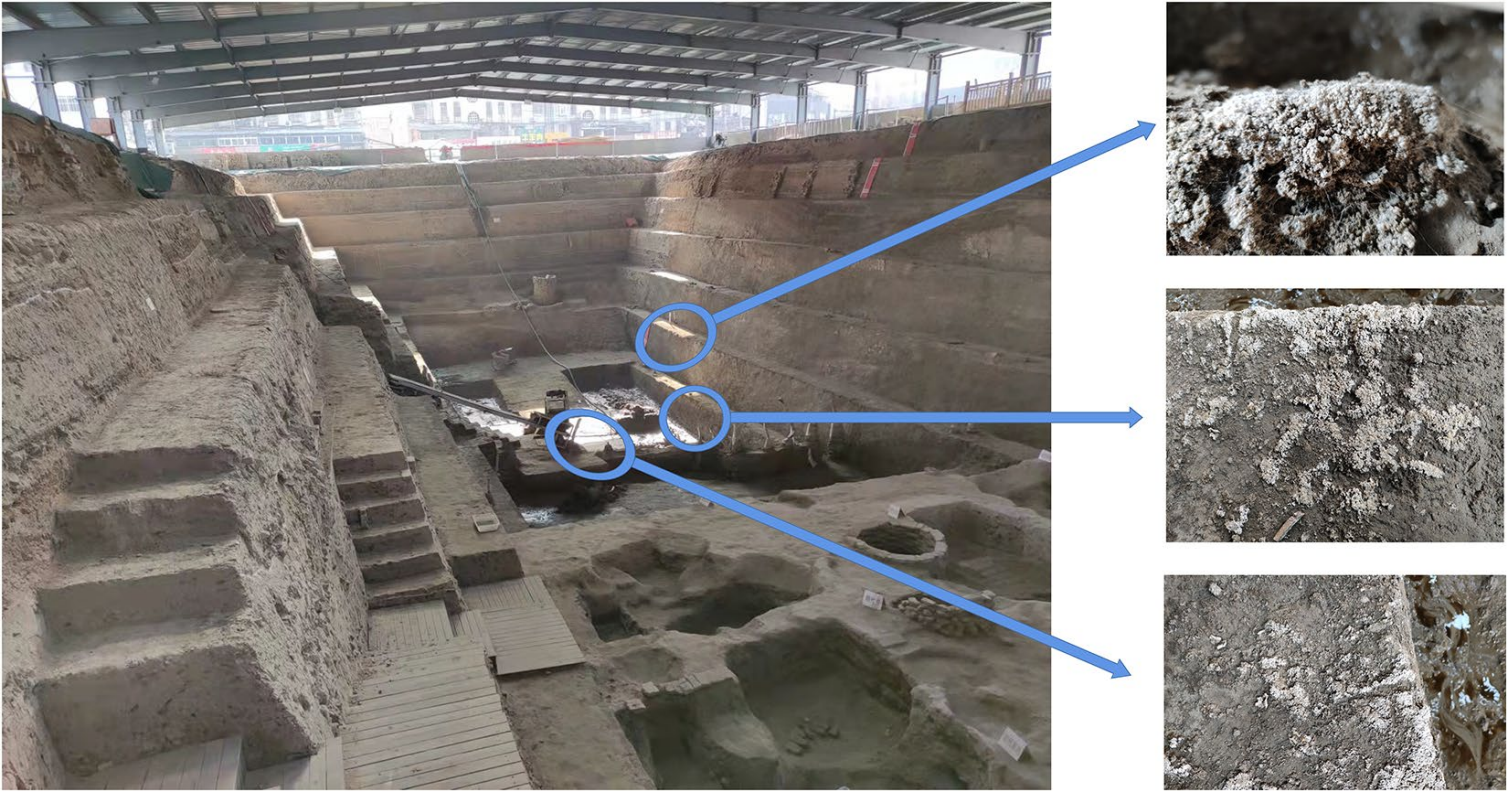
Present situation and salt damage of Zhouqiao site.

Ancient sites with historical, cultural and scientific values with soil as the main construction material, with obvious regional characteristics. As soil sites remain for a long time, they can suffer from different types of diseases, and the typical site is the Zhouqiao site. The excavation of the Zhouqiao site exposed a number of deteriorating diseases in the year or so after the excavation (Fig. 1). Compared with the excavation process of other sites, Zhouqiao site has the characteristics of rapid disease development. The main reason is that it is located in the Yellow River flood area, and the salinization is serious, which makes the salt damage in Zhouqiao site the most serious.

The deterioration of salt on site soil has attracted the attention of many scholars. So far, studies on the effect of salinity on site soil deterioration have focused on the types of salt diseases^1–4^, the distribution patterns of salinity^5–8^ and the mechanisms of site soil deterioration^9–12^. Under the action of salt, the main diseases produced by buildings and soils are flake stripping, salt precipitation, chalking, root hollowing, etc.^13^. These diseases can eventually lead to cracks in the soil or buildings until they collapse. In response to the above literature, most of the earthen sites in the literature are in the northwest arid zone, dry and sandy, with little moisture, and salinization is not as serious as in the yellow panhandle, which is very different from the environment in which the Zhouqiao site is located. At the same time, after the excavation of the Zhouqiao site, its interior was suddenly exposed to the air, which made the evaporation rate of water in the site faster, Moreover, the Zhouqiao site is located in the central urban area, and there is domestic wastewater in its vicinity, and the salt content in the soil body of the yellow floodplain is high, plus the intervention of salt in domestic wastewater, which makes more salt in the soil site and more complicated influencing factors.

In each site, due to the combined effect of moisture, salt and temperature, the salt distribution pattern within the building gradually changes from a "high–low–high" pattern to a "high–low" pattern from top to bottom. There are mainly the following aspects about the mechanism of salt deterioration: ① crystallization pressure; ② hydration pressure; ③ volume expansion pressure; ④ heat change; ⑤ chemical action, etc.^9^. Crystallization pressure is formed when the volume occupied by the saturated solution is less than the volume of crystals precipitated by crystallization plus the volume of saturated solution remaining. The hydration pressure is the pressure generated by the increase in volume of salt caused by hydration. Volume expansion pressure is the pressure generated by the increase of crystallization volume. The volume expansion pressure will generally accompany the crystallization pressure^10^. In some of the above literature, the target audience is different and the environment is different, making some of the theories inapplicable to the Zhouqiao site itself.

There have been many studies on the effect of salinity on the soil itself, but most of them focus on the salt damage itself, and there is less research on the properties of the site soil, and there is a lack of systematic research on the process of salinity, and a lack of theoretical and analytical tools on the micro level of salinity degradation. Because earthen sites are influenced by the environment, and because earthen sites vary greatly from region to region and from era to era, and because the condition of each site itself is different, the study of salinity should be combined with the salt composition and content of earthen sites. In this paper, we use orthogonal tests to simulate the migration of salt in the soil of the Zhouqiao site based on the reality of salt analysis within the site, in order to investigate the mechanism of salt action on the soil of the Zhouqiao site, with respect to its own composition.

## Materials and methods

### Experimental materials

Site soil: The basic physical properties of the soil are shown in Table 1, and the soil column test was conducted using the soil of the Zhouqiao site as the object of study. The size of the soil column was taken as 39.1 mm × 80 mm.

**Table 1:** Basic parameters of soil at Zhouqiao Site.

Liquid limit/%	Plastic limit/%	Plasticity index	Maximum dry density/(g/cm^3^)	Natural moisture content/%
38.5	23.2	15.2	1.519	12.5

Salt: The selection of salt types is based on the salt crystallized from the Zhouqiao site, and the three salts with the highest content are used as the salt solution for this experiment, with the highest content of sodium ions, magnesium ions, calcium ions, chloride ions and sulfate ions. Sodium chloride solution, magnesium sulfate solution and calcium chloride solution with mass fractions of 1%, 3% and 5% were taken as the salt solution used for the test, and pure water was used as the control group.

### Experimental design

In order to investigate the change law of the site soil under the actual working condition, the water content is taken as the natural water content, and the experiment is conducted by orthogonal experimental method with the combination shown in Table 2, where the tenth group is the control group. Place the soil column on the permeable stone and place the permeable stone in the solution, the solution height is about 15 mm from the permeable stone, this data is proportionally derived, site soil height: water table height = soil column height: solution height. After carrying out salt infiltration for ten minutes, take it out and put it into the oven to dry for five hours, infiltration for ten minutes is to ensure that each salt infiltration solution quality is the same and in ten minutes the solution infiltration amount is the same as the wet position of the soil within the site, after drying, observe its surface changes, determine its quality changes, salt analysis out of the height, surface strength, and conductivity, and then analyze it for electron microscope scanning, this is a cycle. The number of cycles is set at 10, 20, and 30 times based on the number of days when the maximum temperature exceeded 25 °C in Kaifeng in the 1970s to 1990s.

**Table 2:** Orthogonal test.

Test no	Control variable			Specimen combination
	Salt type (A)	Salt solution concentration (B)	Drying times (C)	
1	MgSO_4_	1%	10	A_1_B_1_C_1_
2	MgSO_4_	3%	20	A_1_B_2_C_2_
3	MgSO_4_	5%	30	A_1_B_3_C_3_
4	NaCl	3%	10	A_3_B_2_C_1_
5	NaCl	5%	20	A_3_B_3_C_2_
6	NaCl	1%	30	A_3_B_1_C_3_
7	CaCl_2_	5%	10	A_2_B_3_C_1_
8	CaCl_2_	1%	20	A_2_B_1_C_2_
9	CaCl_2_	3%	30	A_2_B_2_C_3_
10	–	–	30	A_0_B_0_C_3_

### Quality test

Some salt will be left in the soil column after each cycle. After each cycle of drying, the mass of the soil column is measured and recorded. If the mass increases with the number of cycles, it means that salt accumulates inside the soil column, and salt accumulation can be expressed by the rate of change of mass.

### Salt analysis test

During the drying process, as the water inside the soil column evaporates into the air, it makes the salt content inside the column higher than the solubility, thus there will be salt crystallization precipitation on the surface of the column. After each cycle of drying, measure the distance of the position analyzed by salt from the bottom surface of the soil column and record.

### Nominal strength test

The nominal strength of the soil column is characterized by the data shown by the drop of one millimeter after the contact between the probe of the push-tension meter (Adelberg digital display push–pull meter hp-2) and the soil column. After the second, fourth, seventh, tenth, fifteenth, twentieth, twenty-fifth and thirtieth cycles, the strength of the surface of the soil column was measured using a push–pull meter, and the four locations of two-thirds of the soil column and the outermost part of the cross-section at that location, the middle of the outermost part and the center of the circle, and the center of the circle were selected as the strength test points. After pressing the probe of the push–pull meter into one millimeter, the peak strength at this point is measured as the strength test result of the soil column.

### Conductivity

Conductivity testing in the form of solutions. The column was divided into three parts, and each part was dissolved in 200 g of pure water and filtered after shaking and dissolving. 100 ml of the filtered liquid was taken and the conductivity of the solution was measured using a conductivity meter (RS485 type).

### Microscopic test

Samples of soil columns dried after the 4th, 10th, 15th and 20th cycles in 3% MgSO4 solution were taken and subjected to SEM electron microscopy (FEI Quanta 250) test. The structural changes inside the specimen before and after treatment and the distribution of salt crystals were observed from a microscopic point of view.

## Results and discussions

### Quality change

**Figure 2:**
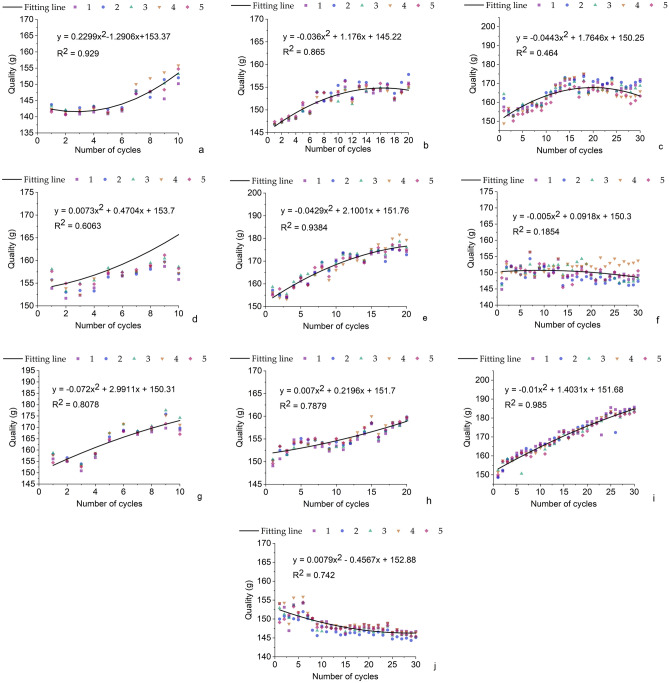
Mass change of each soil column (**a**) Experimental group 1; (**b**) Experimental group 2; (**c**) Experimental group 3; (**d**) Experimental group 4; (**e**) Experimental group 5; (**f**) Experimental group 6; (**g**) Experimental group 7; (**h**) Experimental group 8; (**i**) Experimental group 9; (**j**) Experimental group 10.

The mass change is shown in Fig. 2. As can be seen from Fig. 2, the overall mass of the soil column is in an up-and-down state in each group of tests. In MgSO_4_ solution (Fig. 2a–c), the maximum value of mass is 168.2 g. In NaCl solution (Fig. 2d–f), the maximum value of mass is 179.37 g. In CaCl_2_ solution (Fig. 2g–i), the maximum value of mass is 184.7 g. It can be seen that for salt solutions with a single solute, the increase in salt concentration and the increase in the number of cycles makes the salt accumulate more in the soil column. Comparing the Fig. 2d–f, it can be found that the effect of salt concentration on the mass of the soil column is greater than that of the number of cycles. Meanwhile, it can be seen from Fig. 2j, the mass of each soil column was always constant around a value fluctuating back and forth. This is due to the fact that with the increase of drying times, the salt accumulates in the soil column of the test group due to the continuous absorption of salt, which makes the overall mass of the soil column increase with the increase of cycle times, while there is no salt in the pure water, resulting in a small fluctuation of the mass of the soil column in pure water.

In addition, each graph in the figure shows a trend of rapid growth followed by slow growth. Comparison of the plots shows that the growth of the soil column mass is influenced by the salt concentration as well as the number of cycles. There is a critical value for the mass growth of the soil column, which varies with salinity and salt concentration. The critical value arises due to the high salt concentration in the soil column and the reduction of pore space, which makes salt uptake difficult and eventually leads to lower salt uptake^14^.When this threshold is exceeded, salt uptake will still take place, but at a much reduced rate.

### Salt analysis height

**Figure 3:**
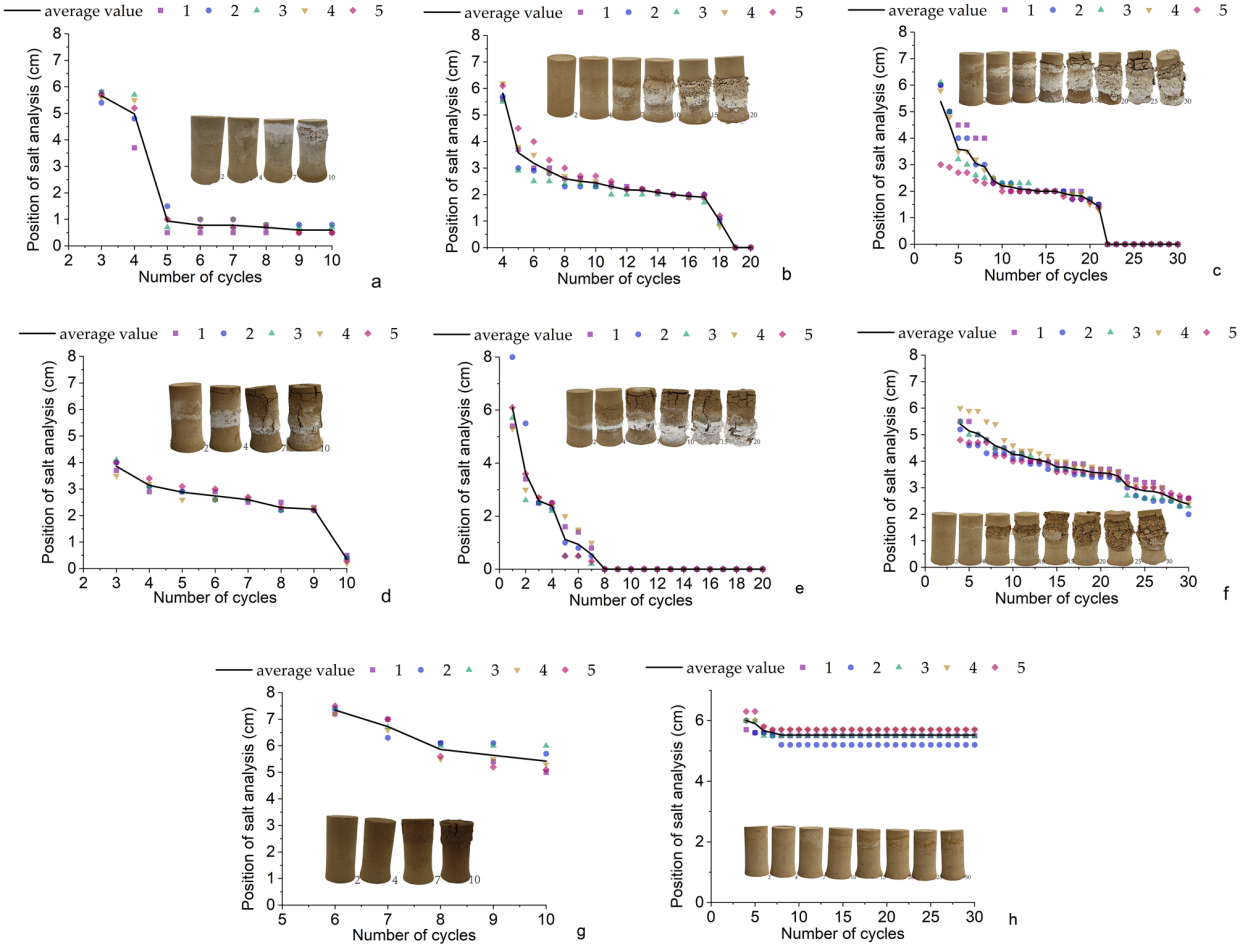
Change of mass and salt position of each soil column: (**a**) Experimental group 1; (**b**) Experimental group 2; (**c**) Experimental group 3; (**d**) Experimental group 4; (**e**) Experimental group 5; (**f**) Experimental group 6; (**g**) Experimental group 7; (**h**) Experimental group 10.

The variation in the height of salt precipitation for different salts is shown in Fig. 3, where the height is the distance from the bottom of the soil column where the salt starts to precipitate. The graph shows that the salt first starts to precipitate from the top of the soil column^15–18^. During the experiment, as the temperature rises the water is gradually evaporated, so that the content of salt in the solution inside the soil column is higher than the solubility, thus causing the salt to crystallize and precipitate^19,20^. From Fig. 3, it can be seen that each salt action causes the soil column to be damaged^21,22^,but they are different in the process of damage for each salt^23^.The soil column in NaCl solution has both cracks and salt analysis^19,24^(Fig. 3d–f). The salt analysis is more serious in MgSO_4_ solution^20^ (Fig. 3a–c) but in CaCl_2_ solution, the cracks are produced more seriously^25^(Fig. 3g). In the control group of pure water, there was no significant salt analysis and no cracks were produced. In Fig. 3, there is a large drop in the curve, and this is the time when salt crystallization spreads over the entire surface of the soil column. In Fig. 3h, there is not very obvious salt analysis out at the beginning, because the soil of the Zhouqiao site already contains part of salt, and in the process of continuous warming and water absorption, it makes part of this salt crystallize and precipitate, because the content of salt in the soil column is limited, so the salt analysis out will not reach the bottom of the soil column.

**Figure 4:**
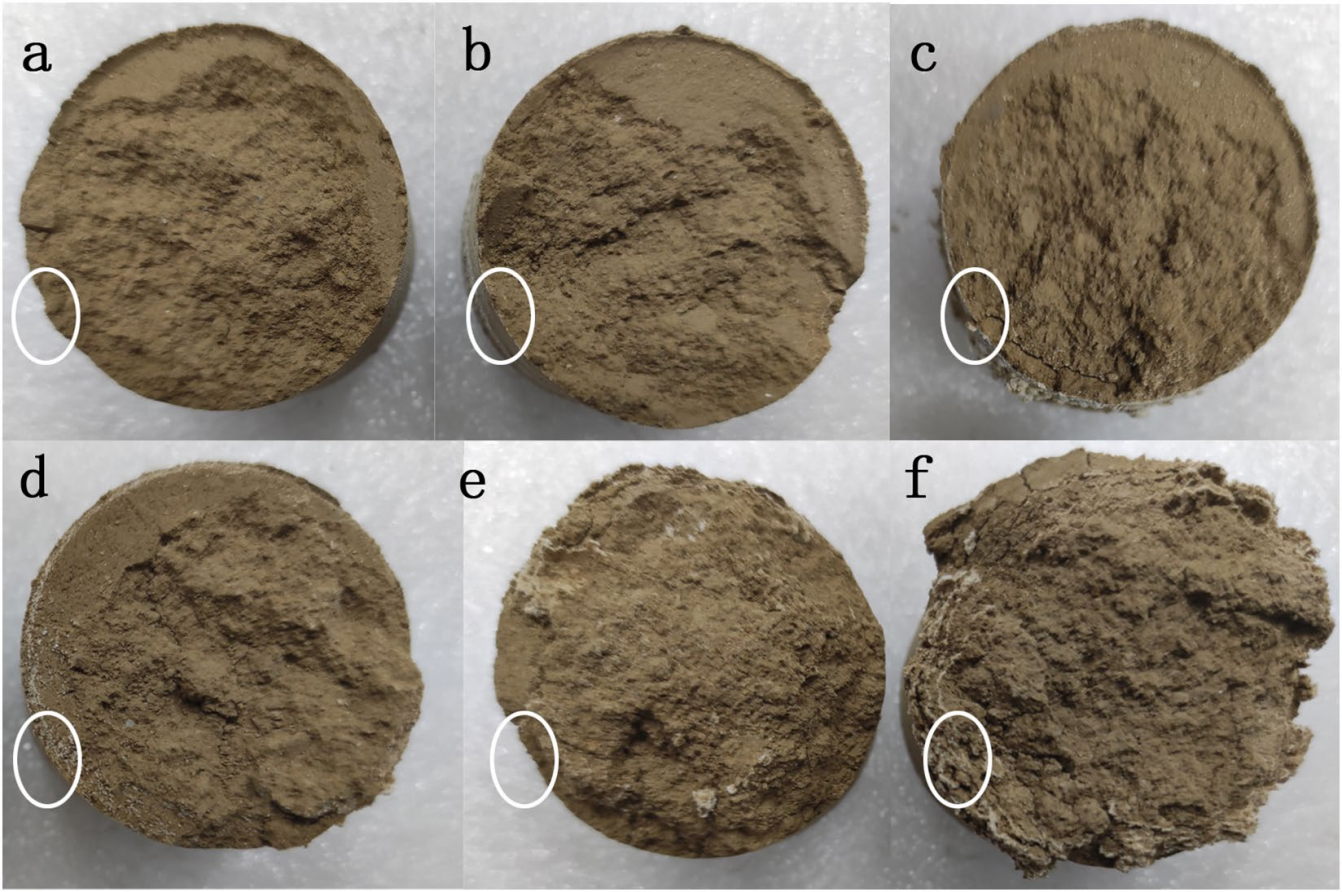
Salt crystallization in soil column with different cycles of 3% MgSO_4_ solution: (**a**) 2nd cycle; (**b**) 4th cycle; (**c**) 7th cycle; (**d**) 10th cycle; (**e**) 15th cycle; (**f**) 20th cycle.

In Fig. 4, it can be seen that in the case of magnesium sulfate solution, the salt precipitation inside the soil column is from outside and inside. The reason for this phenomenon is that within the soil column, salts are transported with water^26,27^, while water migrates from the interior to the exterior of the column under the influence of temperature, which causes a large amount of salts to remain on the surface of the column and thus precipitate in the outermost layer^28^.Next, due to the crystallization of salt in the outermost layer, the dry density of the soil increases and the pore space decreases, so that the salt begins to crystallize and precipitate in the adjacent layer of soil, eventually showing the phenomenon in Fig. 4.

### Nominal strength

**Figure 5:**
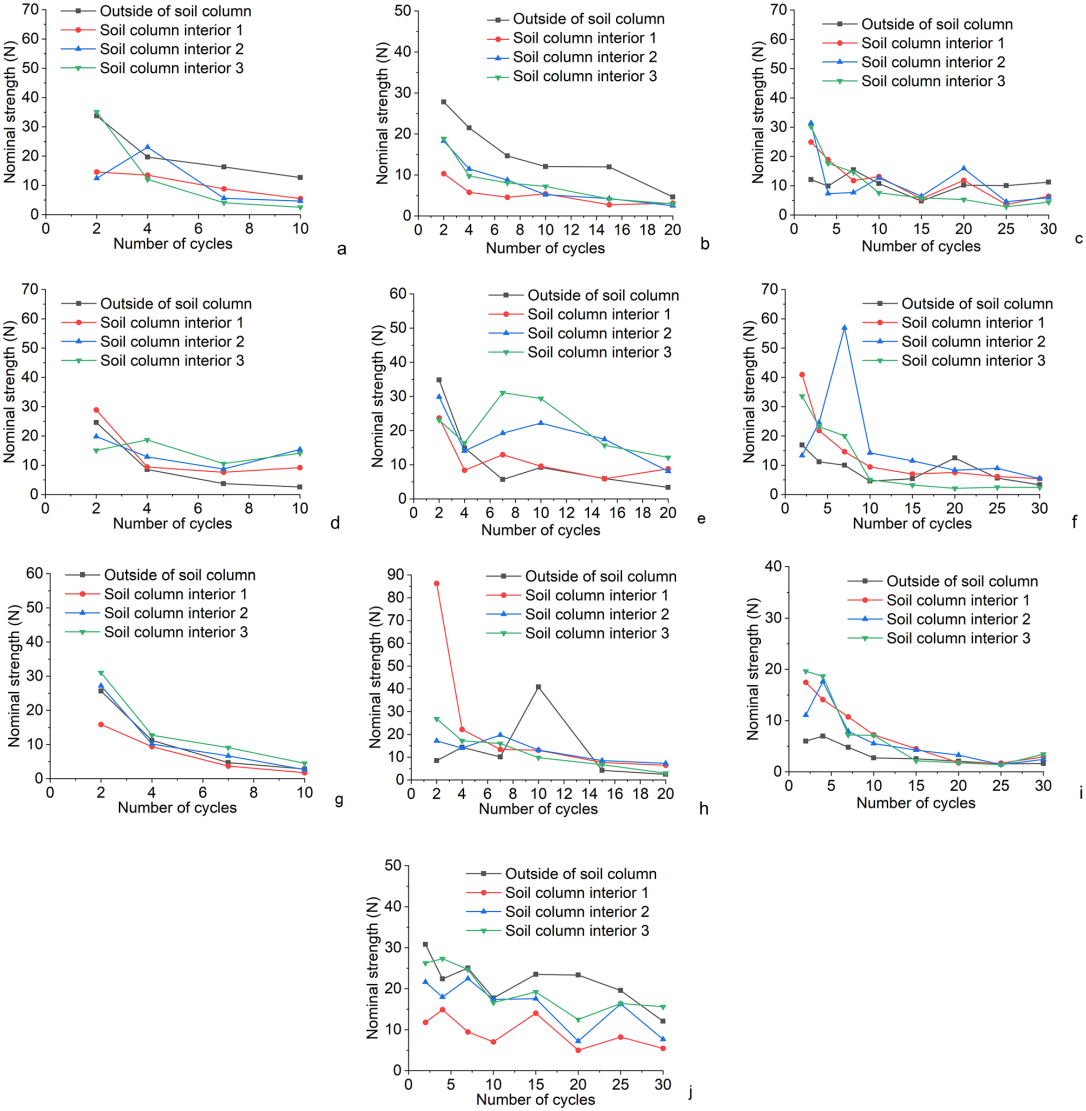
Change of nominal strength of each soil column (**a**) Experimental group 1; (**b**) Experimental group 2; (**c**) Experimental group 3; (**d**) Experimental group 4; (**e**) Experimental group 5; (**f**) Experimental group 6; (**g**) Experimental group 7; (**h**) Experimental group 8; (**i**) Experimental group 9; (**j**) Experimental group 10.

**Figure 6:**
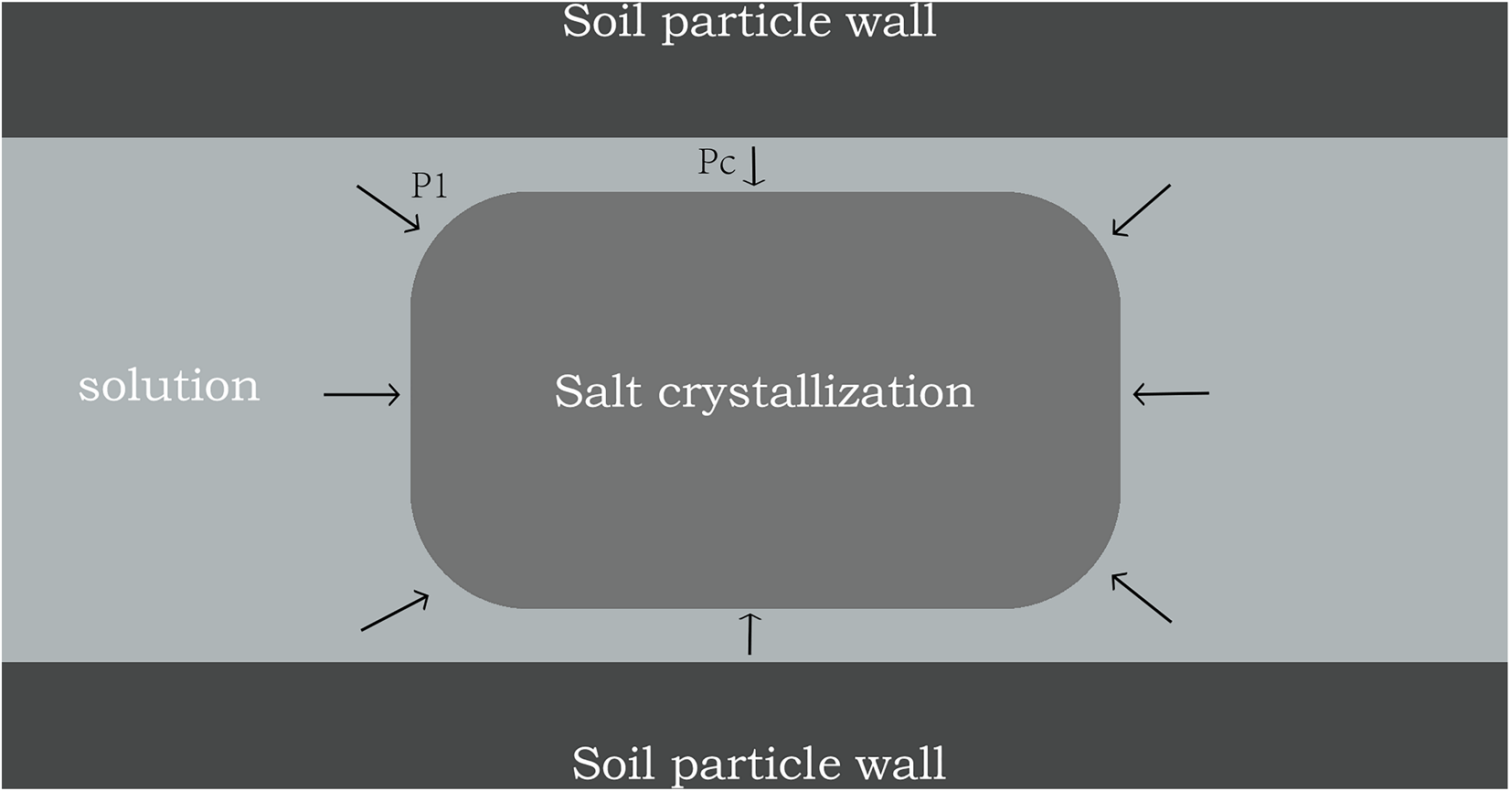
Schematic diagram of salt crystal crystallization between soil particles^34^.

As shown in Fig. 5, the surface strength of each soil column showed a decreasing trend^29^. The internal strength of the soil column occasionally increases due to salt crystallization inside the column, and when the amount of crystallization is small, the crystallization fills the pores between the soil particles, making the soil more dense, and at this time, the strength of the soil column increases. Subsequently, more salt is analyzed, causing the soil particles to be squeezed and then cracked, eventually causing damage to the soil column^30,31^. Salt action until destruction is mainly related to crystallization pressure and capillary action. Under the capillary action, the salt penetrates into the soil column together with the water. During the drying process, the salt crystallizes to produce a crystalline pressure. Correns established an expression for the salt crystallization pressure for a supersaturated pore solution (Fig. 6)^32^.

$$\Delta P{V}_{s}=RT\mathrm{ln}\,\frac{C}{{C}_{s}}$$

where $\Delta P={P}_{C}-{P}_{1}$ is the pressure generated by the crystal; R = 8.3145 MPa cm^3^ mol^−1^ K^−1^ is the ideal gas constant; *T* is the absolute temperature; *V*_s_ is the molar volume of the crystal; *C* and *C*_s_ are the molar mass concentration and saturated molar mass concentration of the current solution, respectively. As the number of cycles increases, the supersaturation ratio of the solution in the pore increases, while the molar volume of the crystal is certain, the pressure generated by crystallization also increases. The increase of crystallization pressure between soil particles makes the connection between particles loose, thus reducing the strength of the soil column.

For a soil column circulating in pure water, only the absorption and evaporation of free water in the column and the dissolution and migration of its own salts are processes that do not result in large crystallization areas. During the test, due to its own gravity factor, the lower part of the soil column produces expansion and deformation, and after repeated water absorption and drying, the microstructure of the soil changes, such as the loss of salt soluble matter in the soil, the development of local tiny fissure channels, etc. weaken the linkage force between soil grains, so that the soil body produces a certain strength reduction effect^33^.

### Conductivity

**Figure 7:**
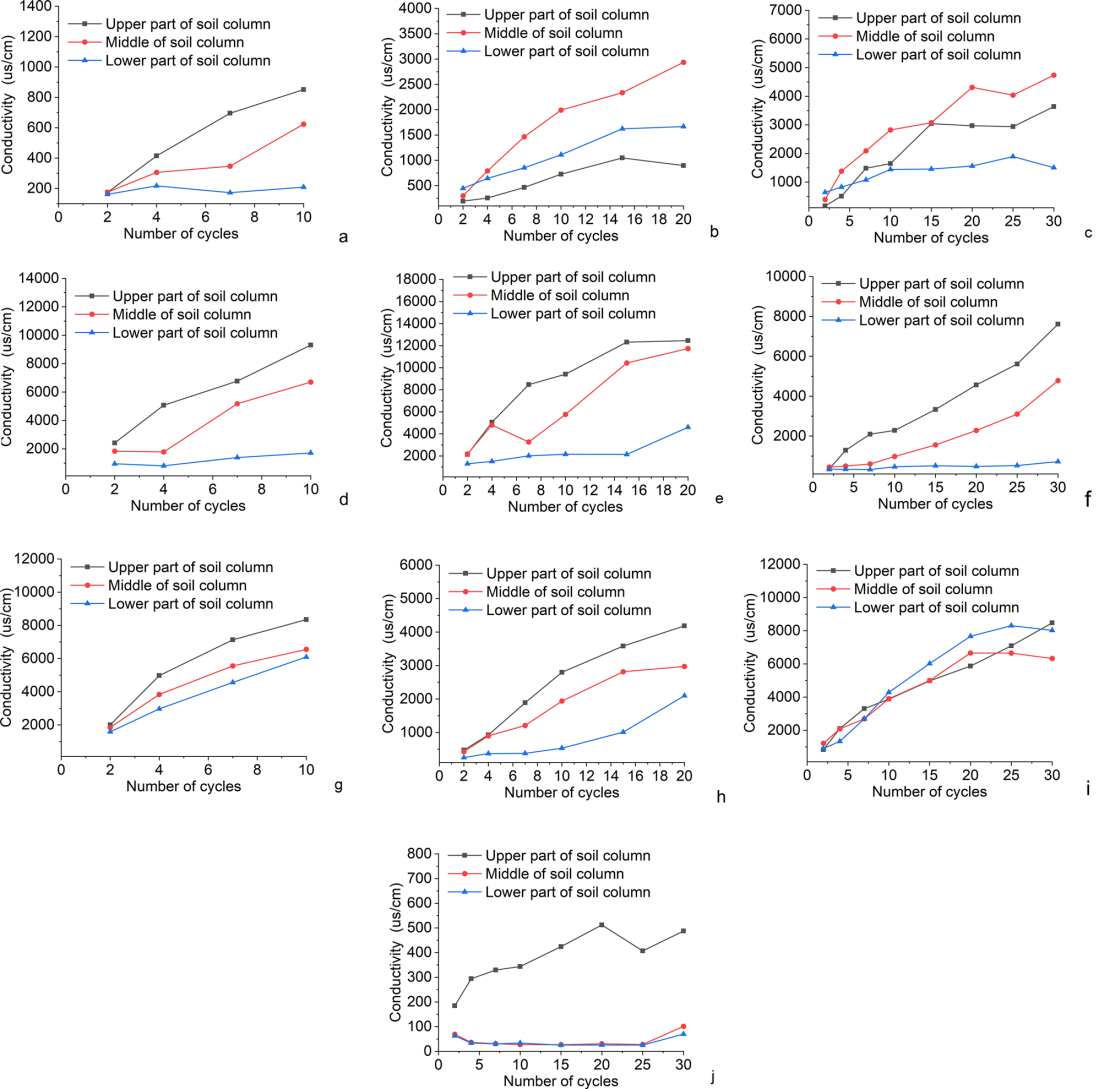
Conductivity change of each soil column (**a**) Experimental group:1; (**b**) Experimental group 2; (**c**) Experimental group 3; (**d**) Experimental group 4; (**e**) Experimental group 5; (**f**) Experimental group 6; (**g**) Experimental group 7; (**h**) Experimental group 8; (**i**) Experimental group 9; (**j**) Experimental group 10.

As shown in Fig. 7, with the increase in the number of cycles, most of the salts in the soil column accumulated in the upper part of the column, and the conductivity of the upper, middle and lower parts of the column decreased sequentially, which also indicated that the migration of salts in the column was from bottom to top^35,36^. In MgSO4 solution (Fig. 7a–c), the maximum value of conductivity is 4735 us/cm. In NaCl solution (Fig. 7d–f), the maximum value of conductivity is 12,454 us/cm. In CaCl2 solution (Fig. 7g–i), the maximum value of conductivity is 8473.67 us/cm. It can be seen that for single solute salt solutions, the soil column conductivity is influenced by the salt concentration and the number of cycles, which increases with increasing salt concentration and the number of cycles. The effect of solution concentration on the conductivity of the soil column was higher than the number of cycles and the permeation rates of the different salts in the column were not the same, with the fastest permeating being NaCl and the slowest being MgSO_4_.

Cai et al^37^. showed that the regression equation of soil salinity (x) and conductivity value (y) in semi-arid saline zone (Henan) was $y=0.002 + 0.291 x$. where *y* is the conductivity value of the soil and *x* is the salinity of the soil. The determination of conductivity is also influenced by ion concentration, ion composition, ion migration rate, solution temperature and conductivity cell constant^38^. As can be seen from the equation, the higher the salinity, the higher the measured value of conductivity^39–41^. In Fig. 7, the conductivity also shows a linear increase situation with the number of cycles, as the number of cycles increases, the salinity within the soil column increases, and the conductivity increases accordingly, in agreement with what is stated in the equation. From Fig. 7j, it can be seen that in pure water, the conductivity of the lower and middle part of the soil column is very low and almost unchanged. This is due to the fact that the ionic content in pure water is almost 0. The salts in the soil column move with the water to the top of the column during the drying process, and then the salt content in the solution is greater than the solubility due to the evaporation of water, which makes the salts accumulate and precipitate in the upper part of the column, thus making the conductivity of the upper, middle and lower parts of the column in pure water show the distribution as shown in the figure.

### SEM result analysis

**Figure 8:**
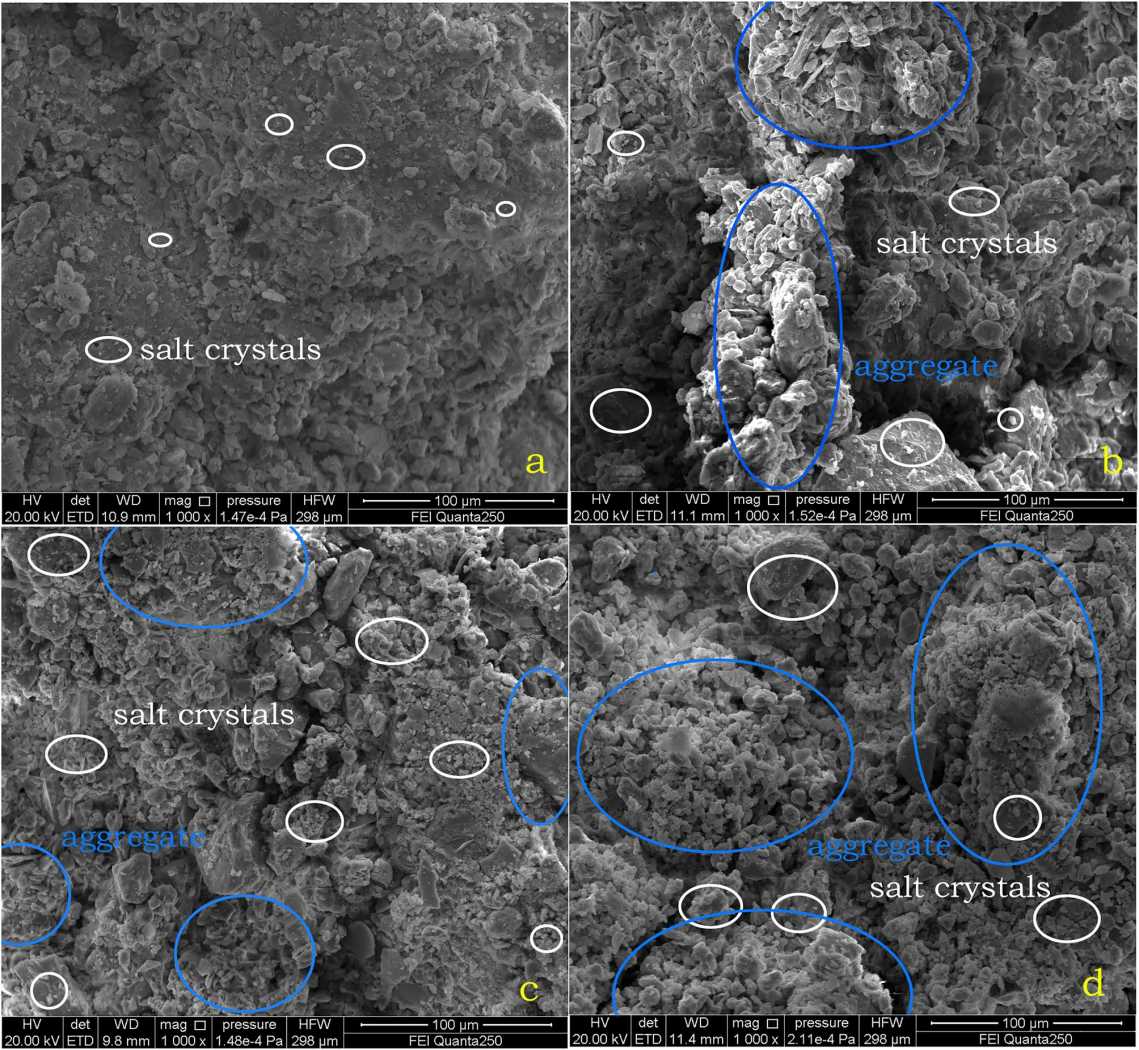
Results of the 4th, 10th, 15th and 20th electron microscope tests of 3% MgSO_4_: (**a**) 4th cycle; (**b**) 10th cycle; (**c**) 15th cycle; (**d**) 20th cycle.

The soil column during the cycling process was partially sampled for scanning electron microscopy, and the microstructure of the soil sample at different cycle times is shown in Fig. 8 (White circles are salt crystals, blue circles are agglomerates^42^, which are not completely circled).From Fig. 8, it can be seen that as the circulation process advances, soil particles undergo significant agglomeration^43^. This is due to the decrease in permeability repulsion and increase in van der Waals forces between soil particles during circulation, which leads to the formation of particles coarser than the soil particles. As the circulation process proceeds, when the adhesion between the particles is dominated by these particles, larger pores are formed, essentially changing the structure of the specimen and making the soil column loose^44,45^.

## Discussions

The amount of absorbed solution in the soil column is the same during the same time interval. But the higher the concentration of the solution, the more the mass of the soil column increases. The increase in mass under all solutions was greater than the increase in mass of the soil column in the pure water condition in the control group. The reasons for this are generally the following. 1. There is a concentration difference between the salt content of the specimen and the salt content of the solution, which allows the solution to diffuse in the specimen. The larger the concentration difference, the stronger the diffusion effect, resulting in more solution into the specimen, thus making the quality of the dried specimen higher. 2. The solution is denser than pure water, thus absorbing or retaining a greater mass of solution in a given volume of pore space. 3. The surface tension of salt solution is higher than that of pure water, thus the capillary migration rate of salt solution is greater than that of pure water. In the process of mass increase, it can be found that after a certain number of cycles the mass increase rate of the soil column decreases significantly, at this time the salt absorption reaches a critical value, the critical value is mainly related to the salt concentration in the soil column, the salt concentration in the soil column is too high, making the diffusion effect lower, also due to the salt crystallization in the soil column makes the pores in the column become dense, so that the infiltration effect also decreases significantly.

At the beginning of the cycle, a certain amount of increase in the strength of the specimen can be found under the action of different types of salts. The reason for this is that as the water penetrates, the soluble salts crystallize in the pores of the soil column, making the soil particles more compact and thus stronger.

At the end of the test, the conductivity showed that the salt distribution in the soil column was decreasing from top to bottom under the action of NaCl and CaCl2, but the largest salt content in the soil column was in the middle of the column under the action of MgSO4, mainly due to the crusting effect of MgSO4 in the crystallization process, which restricted the upward migration of salt, thus causing the salt to be mostly concentrated in the middle of the soil column.

From the electron microscope, it can be found that many large particles can be formed in the soil column. It is mainly due to the agglomeration effect of salt on the fine particles in the soil column, which causes a relative decrease in the proportion of fine particles and a relative increase in the proportion of coarse particles, and thus more coarse particles can be found in the soil column in the electron microscope.

## Conclusion

This paper uses orthogonal tests combined with SEM microscopic tests to investigate the mechanism of salt influence on the soil of the Zhouqiao site, and initially obtained the following understanding:

1. After the wet and dry cycle in the salt solution, the accumulation of salt in the soil column grows rapidly and the surface of the column slowly starts to produce crystallization. When the critical value is reached, the absorption rate of the column under the salt decreases greatly, but the destruction rate of the column starts to become faster at this time. In the cross-section of the soil column, the salt first crystallizes on the outermost side. In the cross section of the soil column, the salt first crystallizes on the outermost side, and when the crystallization reaches a certain amount, the outer pore space is reduced, and the subsequent salt will crystallize in the adjacent layer of soil, eventually forming the phenomenon of salt precipitation from the outside to the inside.
2. At the beginning, the salt crystals within the soil column will serve to fill the pores, densify and strengthen the soil column, making it stronger, but when the pores are filled, the salt crystals will continue to grow enriched within the pores and cause damage to the soil column. Salinity causes agglomeration of fine particles in the soil column, resulting in coarser particles, thus forming larger pores, changing the structure of the specimen and reducing the strength of the soil column.
3. Capillary action causes the migration and diffusion of salt within the site soil, and after repeated cycles, the recrystallization of salt within the site soil, and the recrystallization process causes more obvious damage to the site soil, and in the process of destruction it causes the abatement of the soil site and affects the preservation of the soil site.
4. Salt damage to earthen sites requires water as a solute. Salt migrates with water in earthen sites and crystallizes with the evaporation of water, so it is more important to focus on the control of precipitation and groundwater in the conservation process of earthen sites to reduce salt damage to earthen sites.

## Data Availability

The test steps can be repeated. The data and material obtained are true and effective. The data used to support the findings of this study are available from the corresponding author upon request.
